# The investigation of a traditional Chinese medicine, Guizhi Fuling Wan (GFW) as an intravesical therapeutic agent for urothelial carcinoma of the bladder

**DOI:** 10.1186/1472-6882-13-44

**Published:** 2013-02-23

**Authors:** Chi-Chen Lu, Mei-Yi Lin, Syue-Yi Chen, Cheng-Huang Shen, Lih-Geeng Chen, Hsiao-Yen Hsieh, Michael WY Chan, Cheng-Da Hsu

**Affiliations:** 1 Department of Chinese Medicinec, Ditmanson Medical Foundation Chia-Yi Christian Hospital, Chia-Yi, Taiwan; 2 Department of Medical Research, Ditmanson Medical Foundation Chia-Yi Christian Hospital, 539 Zhongxiao Road, 600, Chiayi City, East District, Taiwan; 3 Department of Urology, Ditmanson Medical Foundation Chia-Yi Christian Hospital, Chia-Yi, Taiwan; 4 Graduate Institute of Molecular Biology, National Chung Cheng University, 168 University Road, Chia-Yi, Min-Hsiung, Taiwan; 5 Department of Life Science, National Chung Cheng University, Room 452, 168 University Road, Chia-Yi, Min-Hsiung, Taiwan; 6 Department of Microbiology, Immunology and Biophamaceuticals, National Chiayi University, Chiayi, Taiwan

## Abstract

**Background:**

The high risk of recurrence faced by patients with bladder cancer has necessitated the administration of supplemental intravesical chemotherapy; however, such treatments often result in severe side effects. As a result, novel intravesical agents with enhanced efficacy and minimal toxicity are urgently required for the treatment of bladder cancer.

**Methods:**

Guizhi Fuling Wan (GFW) is a traditional Chinese medicine shown to inhibit the growth of hepatocellular carcinoma. This study evaluated the growth inhibition of GFW using normal human urothelial cells and bladder cancer cells; the efficacy of GFW treatment was further compared with mitomycin C, epirubicin, and cisplatin. We also examined the progression of cell cycle and apoptosis in bladder cancer cells in response to GFW treatment. CCK-8 was employed to analyze cell viability and flow cytometry was used to study the cell cycle and apoptosis. The mechanisms underlying GFW-induced cell cycle arrest were determined by Western blot analysis.

**Results:**

Our data demonstrate the potent inhibitory effect of GFW in the proliferation of bladder cancer cell lines, BFTC 905 and TSGH 8301. GFW presented relatively high selectivity with regard to cancer cells and minimal toxicity to normal urothelial cells. Our results also demonstrate that GFW interferes with cell cycle progression through the activation of CHK2 and P21 and induces apoptosis in these bladder cancer cells.

**Conclusions:**

Our results provide experimental evidence to support GFW as a strong candidate for intravesicle chemotherapy against bladder cancer.

## Background

Urothelial carcinoma (UC) is the most common cancer of the urinary tract [1] and the fourth most common malignancy in the U.S. [2]. The most common histological type of UC is transitional cell carcinoma (TCC), which is originated from the urothelial lining of the urinary tract [3]. Although UC may occur anywhere in the urinary tract, it generally originates in the urinary bladder [4]. Bladder cancer patients diagnosed with non-muscle invasive disease have a high risk of recurrence [5], necessitating the use of intravesical chemotherapy or Bacillus Calmette-Guerin (BCG) as a supplement to transurethral resection (TUR) [6]. Unfortunately, intravesical chemotherapy, such as mitomycin C or thiotepa, commonly produce severe side effects, including urinary frequency, urinary urgency, cystitis, and hematuria [7]. Thus, novel intravesical agents with proven efficacy and minimal toxicity are urgently required for the treatment of bladder cancer.

Guizhi Fuling Wan (GFW) is a well-known traditional Chinese herbal formula, comprising five herbs including Cinnamomi Ramulus, Poria Cocos, Paeoniae Radix Rubra, Persicae Semen, and Moutan Cortex [8]. It has been used extensively throughout Asia in the treatment of blood stasis [9-11]. Due to its sedative, analgesic, and anti-inflammatory effects, GFW has also been used in the treatment of various diseases. For example, GFW has been shown to inhibit the growth of hepatocellular carcinoma [12,13] and cervical cancer [14]. However, the effect of GFW on urothelial carcinoma has never been explored. This study compared the effects of GFW with various other chemotherapeutic agents in the growth of normal human urothelial cell and two cancer cell lines. The effects of these agents on cell cycle progression and apoptosis in urothelial cancer cells were also compared. Finally, we sought to reveal the underlying mechanisms involved in cell cycle arrest induced by GFW.

## Methods

### Preparation of agents and cell cultures

GFW herbal extract (batch No. 221141) was purchased from Sun Ten Pharmaceutical Co., Ltd. (Taichung City, Taiwan) and validated using HPLC as outlined in the Supplemental Experimental Procedures (see Additional file 1). GFW and Ramulus Cinnamomi (otherwise known as Guizhi) (Sun Ten Pharmaceutical, Taiwan, ROC) were dissolved in ddH_2_O and filtered using a 0.22 micron filter at 220 mg/ml and 129.4 mg/ml, respectively. The concentrations of these stock solutions were then confirmed by weighing after lyophilization. Mitomycin-C (Kyowa Hakko Kirin Co., Tokyo, Japan), Epirubicin (Actavis Italy S.P.A., Milano, Italy) and Cisplatin (ABIC Biological Laboratories Ltd., Netanya, Israel) were dissolved in normal saline buffer at 1 mg/ml to provide stock solutions which were then diluted with cell culture medium to desired concentrations ranging from 0.0025 to 0.08 mg/ml. Human bladder papillary transitional cells, BFTC 905, and bladder carcinoma cells, TSGH 8301 (Food Industry Research and Development Institute, Taiwan, ROC) as well as primary normal urothelial cells, HUC 4449 (ScienCell Research Laboratories, Carlsbad, CA, USA) were used as cell models. BFTC 905 and TSGH 8301 were cultured in RPMI 1640 supplemented with 10% fetal bovine serum (FBS), 100 units/ml penicillin and 100 μg/ml streptomycin. HUC 4449 was cultured in urothelial cell medium (ScienCell Research Laboratory, Carlsbad, CA, USA) supplemented with 10% FBS, 100 units/ml penicillin and 100 μg/ml streptomycin. All cell lines were cultured at 37°C under a humidified atmosphere containing 5% CO_2_. All studies involving human cell lines were conformed to the Helsinki Declaration and approved by the Institutional Review Boards of the Chia-Yi Christian Hospital (reference number: 099078).

### Cell viability assay

BFTC 905, TSGH 8301 and HUC 4449 were initially seeded in 96-well plates at 1 × 10^4^ cells per well and cultured for 24 h. The cells were subsequently starved in medium supplemented without FBS for 24 h, and then treated with the agents of interest at various concentrations for 24 h. Cell viability was then determined using the Cell Counting Kit-8 (CCK-8) (SIGMA, Switzerland), in accordance with the manufacturer’s protocol. In brief, the assay was performed using WST-8, [2-(2-methoxy-4-nitrophenyl)-3-(4-nitrophenyl)-5- (2, 4-disulfophenyl)-2H-tetrazolium, monosodium salt], which can be bio-reduced by cellular dehydrogenases to an orange formazan product, which is then dissolved in cell culture medium. The production of formazan occurs only in living cells at a rate proportional to the number of living cells. After incubating the cells with CCK-8, the light absorbance of the culture medium in each well was measured at 450/655 nm using Microplate reader Model 680 (BIO-RAD). The cell viability rate, compared to the control (untreated cells), was calculated using the following equation.

$$\begin{array}{l} \mathrm{Viability}\left(\%\right)=100\times \mathrm{Absorbance}\;\mathrm{of}\;\mathrm{treated}\;\mathrm{group}\\ \;/\mathrm{Absorbance}\;\mathrm{of}\;\mathrm{untreated}\;\mathrm{group} \end{array}$$

The percentage of viability versus the concentration of a given agent was used to calculate the concentration that would return cell viability of 50% (IC_50_).

The selectivity index (SI), cytotoxic selectivity of the agent against cancer cells, versus normal cells [15], was calculated from the IC_50_ as follows:

$$\begin{array}{l} \mathrm{SI}=IC_{50}\mathrm{of}\;\mathrm{the}\;\mathrm{given}\;\mathrm{agent}\;\mathrm{in}\;\mathrm{normal}\;\mathrm{cells}\\ \;/IC_{50}\mathrm{of}\;\mathrm{the}\;\mathrm{given}\;\mathrm{agent}\;\mathrm{in}\;\mathrm{cancer}\;\mathrm{cells} \end{array}$$

### Cell cycle analysis

BFTC 905, TSGH 8301, and HUC 4449 were initially seeded in 6-well plates at 2 × 10^5^ cells per well and cultured for 24 h. The cells were then starved in medium supplemented without FBS for 24 h, and then treated with 0.5 and 1 mg/ml of GFW for 24 h. Following treatment with GFW, single-cell suspensions were prepared using trypsinization and resuspension in PBS and then fixed with methanol at 4°C overnight. The fixed cells were rehydrated and washed twice with PBS before being stained via incubation with 5 μg /ml propidium iodide (SIGMA, St. Gallen, Switzerland) and 1 mg/ml RNase A for 30 min at room temperature. The cells were then analyzed using a BD FACSCanto II flow cytometer (BD Biosciences, Franklin Lakes, NJ, USA) using Mod Fit LT™ 3.3 software.

### Apoptosis analysis

Cell apoptosis was analyzed using the Annexin V-FITC Apoptosis Detection Kit (BioVision, Mountain View, CA, USA), in accordance with the manufacturer’s protocol. In brief, BFTC 905, TSGH 8301, and HUC 4449 cells were seeded in 6-well plates at 2 × 10^5^ cells per well and cultured for 24 h. They were then treated with 0.5 or 1 mg/ml of GFW for 24 h. Single-cell suspensions were then prepared by trypsinization and resuspended in 500 μl of binding buffer supplied by the manufacturer. The cell suspensions were combined with 5 μl of annexin V-FITC and 5 μl of propidium iodide and incubated at room temperature for 5 min in the dark, before being analyzed using a BD FACSCanto II flow cytometer (BD Biosciences, Franklin Lakes, NJ, USA) using Mod Fit LT™ 3.3 software.

### Western blot analysis

BFTC 905 and TSGH 8301 were seeded in 6-well plates at 2 × 10^5^ cells per well and cultured for 24 h. The cells were starved in medium supplemented without FBS for 24 h, and then treated with 2 mg/ml of GFW for various durations. Cell total proteins were extracted and identified using a Bio-Rad protein assay (Bio-Rad, Hercules, CA) with bovine serum albumin (BSA) as a standard. Each lysate (10 μg) was resolved on denaturing polyacrylamide gels and transferred electrophoretically to a PVDF transfer membrane. After blocking with 3% blocker (Bio-Rad) in TBS-Tween 20 (TBST), the membranes were incubated with primary antibodies [CHK2, 1 : 1000; Phospho-CHK2 (Thr68), 1 : 1000 and p21, 1 : 1000 (Cell Signaling, Danvers, MA, USA)] at room temperature for 2 h. Immunoreactive proteins were incubated with horseradish peroxidase (HRP)-conjugated secondary antibodies for 1 h at room temperature. After being washed with TBST, the reactants were developed using the enhanced chemiluminescence kit (GE Healthcare Biosciences) and identified using the BioSpectrum 800 system (UVP).

### Statistical analysis

The data were expressed as either mean ± SEM or a percentage, relative to the untreated control. Data discrepancies between the treated and untreated control groups were analyzed using one way ANOVA followed by Dunnett’s test. Statistical analysis was considered significant if *P* is less than 0.01 and within the 99.9% confidence interval.

## Results

### HPLC analysis of Guizhi Fuling Wan (GFW)

**Figure 1:**
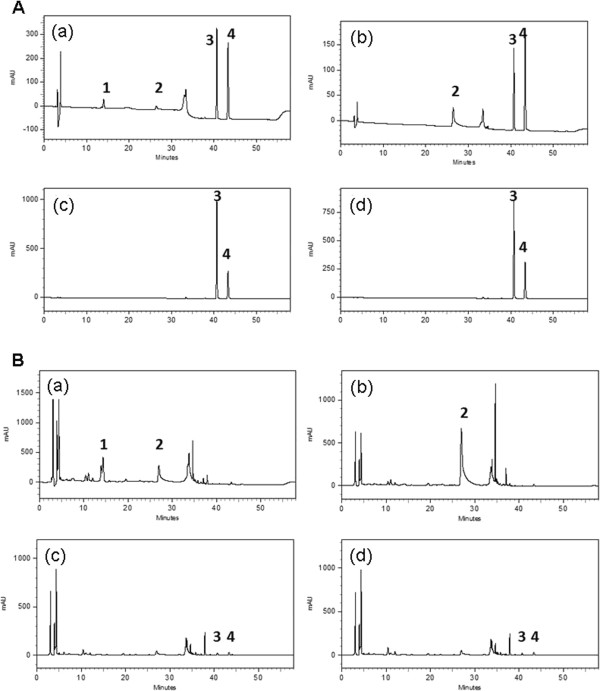
HPLC analysis: (A) HPLC chromatograms of (1) amygdalin, (2) paeoniflorin, (3) cinnamaldehyde, and (4) paeonol detected at (a) 215 nm, (b) 232 nm, (c) 280 nm, (d) 275 nm; (B) HPLC chromatograms of (1) amygdalin, (2) paeoniflorin, (3) cinnamaldehyde, and (4) paeonol in GFW detected at (a) 215 nm, (b) 232 nm, (c) 280 nm, (d) 275 nm.

Guizhi Fuling Wan is composed of five Chinese herbs, including Poria cocos, for which no accepted water-soluble bioactive component is currently available. The bioactive components of Cinnamomi Ramulus, Paeoniae Radix Rubra, Persicae Semen, and Moutan Cortex are cinnamaldehyde, paeoniflorin, amygdalin, and paeonol, respectively. The structural variety of these four compounds produces various absorption wavelengths (see Additional file 1: Figure S1). A photodiode array detector provided simultaneous detection of the compounds as follows: amygdalin (215 nm), paeoniflorin (232 nm), cinnamaldehyde (280 nm), and paeonol (275 nm) (Figure 1A). The calibration equations and correlation coefficients revealed linear relationships between the peak areas and concentrations (see Additional file 1: Table S1). The relative standard deviation (RSD) of these four compounds in GFW fell between 1.54-3.27% (intra-day) and 1.17-2.34%, (inter-day) (Table 1), indicating the high degree of reproducibility associated with this method. The contents of the compounds in GFW were as follows: amygdalin (11.53 mg/g), paeoniflorin (30.26 mg/g), cinnamaldehyde (0.10 mg/g), and paeonol (0.28 mg/g) (Table 1, Figure 1B).

**Table 1:** Contents of amygdalin, paeoniflorin, cinnamaldehyde, and paeonol in GFW with intra-day and inter-day variability

**Compound**	**Intra-day variability**		**Inter-day variability**	
	**Mean ± S.D. (mg / g)**	**RSD (%)**	**Mean ± S.D. (mg / g)**	**RSD (%)**
Amygdalin	11.53 ± 0.18	1.54	11.56 ± 0.14	1.17
Paeoniflorin	30.26 ± 0.99	3.27	30.54 ± 0.69	2.24
Cinnamaldehyde	0.10 ± 0.00	2.40	0.10 ± 0.00	2.34
Paeonol	0.28 ± 0.01	2.69	0.28 ± 0.01	2.31

n=3 for intra-day, n=9 for inter-day.

### Maximum tolerated dosage of GFW in normal urothelial cells

**Figure 2:**
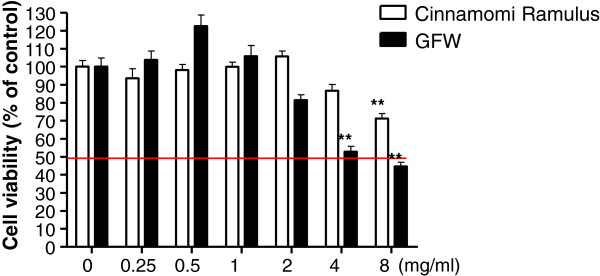
**Cytotoxicity of GFW and Cinnamomi Ramulus against normal human urothelial cell line, HUC 4449: Cells were initially seeded in 96-well plates at 1 × 10**^**4**^**cells per well and cultured for 24 h.** The cells were subsequently starved in medium supplemented without FBS for 24 h, and then treated with various concentrations of agents for 24 h. Cell viability was detected using the Cell Counting Kit-8 (CCK-8). Data are presented as mean ± SEM (n=6). Significant differences from the no treatment control is indicated by * * (p<0.01), as determined by one-way ANOVA and Dunnett’s comparison test.

A fundamental requirement of intravesical agents is minimal cytotoxicity toward normal cells. To determine the maximum dosage of GFW that normal urothelial cells could tolerate, normal human urothelial HUC 4449 cells were treated with various concentrations of GFW and its major component, Ramulus Cinnamomi (Guizhi). The non-toxic effects of Guizhi have been demonstrated in MRC-5 normal lung epithelial cells [16]; therefore, we selected Guizhi as a negative control for this study. According to CCK-8 assay, GFW and Guizhi caused significant death (p<0.01) of HUC 4449 cells at concentrations of 4 mg/ml and 8 mg/ml, respectively, compared to the untreated control (Figure 2). This suggests that GFW and Guizhi have cytotoxic effects on normal urothelium only at high concentrations. We therefore calculated that a concentration of 2 mg/ml would be the maximum safe dose that normal urothelial cells could withstand. This value was applied to all subsequent experiments dealing with cell cycle and apoptosis.

### Cytotoxic effects of GFW, cisplatin, epirubicin, and mitomycin-C on normal urothelial cells and bladder cancer cell lines

**Figure 3:**
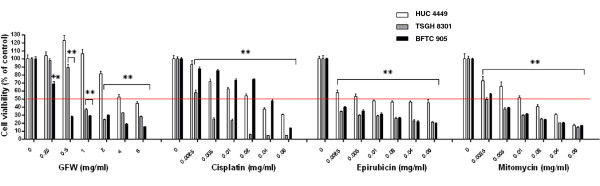
**Cytotoxicity of GFW, cisplatin, Epirubicin and mitomycin against normal human urothelial cell line, HUC 4449 and bladder cancer cell lines, TSGH 8301 and BFTC 905: Cells were initially seeded in 96-well plates at 1 × 10**^**4**^**cells per well and cultured for 24 h.** The cells were subsequently starved in medium supplemented without FBS for 24 h and then treated with various concentrations of agents for 24 h. Cell viability was detected using the Cell Counting Kit-8 (CCK-8). Data are presented as mean ±SEM (n=6). Significant differences from the no treatment control is indicated by * * (p<0.01), as determined by one-way ANOVA and Dunnett’s comparison test.

Next, we performed an *in vitro* cell viability assay to compare the cytotoxicity of GFW with traditional chemotherapeutic agents (mitomycin C, epirubicin, and cisplatin) in normal urothelial and cancer cell lines. Compared with the untreated control, the CCK-8 assay revealed that the cytotoxicity of GFW to TSGH 8301 and BFTC 905 urothelial cancer cells is comparable to that of the three other chemotherapeutic agents with different IC_50_ values (P< 0.001, one-way ANOVA) (Table 2 and Figure 3). Traditional chemotherapeutic agents have much lower IC_50_ towards TSGH 8301 and BFTC 905 cancer cells; however, even such low doses are toxic to normal urothelial cells (Table 2 and Figure 3). Conversely, GFW is only toxic towards cancer cells, demonstrating a high selectivity index (SI) value toward TSGH 8301 cells (8.35) and BFTC 905 cells (19.98). The SI values of GFW are much higher than those of traditional chemotherapeutic agents (Table 2).

**Table 2:** Cytotoxic activities of agents

	**IC**_**50**_**(mg/ml) ± standard deviation**			**Selectivity index (SI**^**c**^**)**	
	**HUC 4449**	**TSGH 8301**	**BFTC 905**	**TSGH 8301**	**BFTC 905**
Guizhi^a^	Inactive^b^	Inactive^b^	Inactive^b^		
GFW	7.1191±0.9628	0.8524±0.1125	0.3564±0.0438	8.35	19.98
Cisplatin	0.0232±0.0069	0.0041±0.0013	0.0440±0.0020	5.66	0.52
Epirubicin	0.0054±0.001	0.0019±0.0002	0.0021±0.0001	2.84	2.57
Mitomycin-C	0.0102±0.002	0.0040±0.0005	0.0041±0.0002	2.55	2.49

Data are expressed as the mean of *n* replicates (n≧6).

^a^ Non-anticancer agent and negative control.

^b^ IC_50_ > 8 mg/ml is considered to be inactive.

^c^ SI value > 3 indicates high selectivity [9].

### GFW arrests cell cycle progression in human bladder cancer cells

**Figure 4:**
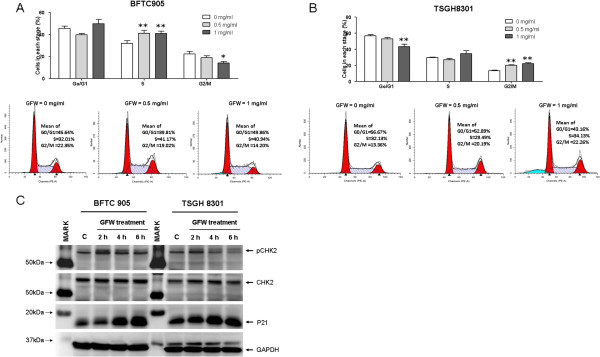
**Effects of GFW on cell cycle of bladder cancer cells: (A) BFTC 905 and (B) TSGH 8301 cells.** Cells were cultured in 6-well plates at 2 × 10^5^ cells per well for 24 h, followed by starvation in medium without FBS for 24 h. The cells were then treated with 0, 0.5, or 1 mg/ml of GFW for 24 h. Cells were harvested and stained using propidium iodide staining solution for 30 min in the dark and analyzed by flow cytometry. Data are presented as the mean ± SEM (n = 4). Significant differences from the no treatment control are indicated by * * (p<0.01), as determined by one-way ANOVA and Dunnett’s comparison test. (**C**) Western blots of phosphorylated CHK2, CHK2, and P21. The expression levels of phosphorylated CHK2 increased in both BFTC 905 and TSGH 8301 cells following treatment with GFW for 2 h. A substantial increase in p21 was observed in BFTC 905 cells following treatment with GFW for 4–6 h. A high basal level of P21 prior to treatment and a mild, gradual increase in p21 level following treatment with GFW for 6 h was observed in TSGH 8301 cells.

We further investigated the effects GFW on the cell cycle progression of urothelial cancer cells. Cell cycle analysis using flow cytometry demonstrated that treating BFTC 905 cells with GFW at concentrations of 0.5 or 1 mg/ml for 24 h would result in significant cell cycle arrest in the S phase (*p* < 0.01, Figure 4A); a concentration of 1 mg/ml resulted in a decrease in the number of cells in the G2/M phase. In contrast, identical treatment of TSGH 8301 cells with GFW resulted in significant cell cycle arrest in the G2/M phase (*p* < 0.01, Figure 4B); a concentration of 1 mg/ml resulted in a decrease in the number of cells in G0/G1 phase.

These results suggest that GFW leads to the cell cycle arrest at the G1 to S transition of BFTC 905 cells and at the S to G2/M transition in TSGH 8301 cells. Accordingly, the increase in the number of BFTC 905 cells in S phase was significant at 0.5 and 1 mg/ml of GFW (p<0.01), which correlates with a reduction in the number of cells in G2/M phase for these concentrations. Conversely, the increase in the number of TSGH 8301 cells in G2/M phase was significant at 1 mg/ml of GFW (p<0.01), which correlates with a reduction in the number of cells in G0/G1 phase at this concentration (p<0.01). We also observed that, compared with the control, treating BFTC 905 and TSGH 8301 with GFW at a concentration of 1 mg/ml for 24 h induced cell apoptosis, as evidenced by the presence of a subG1 population (Figure 4A, B).

To reveal the molecular mechanisms underlying the effects of GFW, we investigated the expression of several cell cycle regulatory proteins. Western blot results show that the expression of phosphorylated Cell Cycle Checkpoint Kinase-2 (CHK2) (Phospho-CHK2) increased significantly following treatment with GFW for 2 h, which was followed by a substantial increase in p21 in BFTC905 cells after treatment for 4–6 h. In contrast, only a slight, gradual increase was observed in the p21 in TSGH8301 cells following treatment for 6 h (Figure 4C). These results indicate that the CHK2/P53/P21 pathway might be involved in the cell-cycle arrest induced by GFW.

### GFW-induced apoptosis in human bladder cancer cells

**Figure 5:**
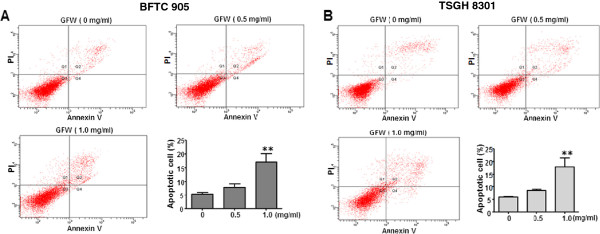
**Effects of GFW on apoptosis of bladder cancer cells: (A) BFTC 905 and (B) TSGH 8301 cells were treated with GFW at concentrations of 0.5 and 1 mg/ml for 24 h.** Cells were then analyzed by FACS to determine the relative% of apoptotic Annexin V/PI cells. The lower left quadrant (Q3) represents viable cells; the upper left quadrant (Q1) represents necrotic cells; the lower right quadrant (Q4) represents early apoptotic cells; and the upper right quadran (Q2) represents nonviable late apoptotic cells. The relative percentages of apoptotic cells including early and late apoptotic cells were determined. Data are represented as mean ± SEM of two independent experiments performed in triplicate. Significant differences from the no treatment control are indicated by * * (p<0.01), as determined by one-way ANOVA and Dunnett’s comparison test.

In light of the previous findings, we further investigated the effect of GFW on the apoptosis of bladder cancer cells. This represents the pharmaco-dynamic endpoint of the actions of anticancer drugs [17] and an autonomous detachment process in which damaged cells are removed, thereby avoiding the inflammatory response normally associated with necrosis and the resulting cytotoxicity to surrounding cells [18]. To investigate the cytotoxic effects of GFW on apoptosis, we treated bladder cancer cells with GFW at concentrations of 0.5 and 1 mg/ml for 24 h followed by analysis using the AnnexinV-FITC staining method, as measured by flow cytometry. Our results presented a dose-dependent increase in apoptosis in cells treated with GFW with significant apoptosis observed at a concentration of 1 mg/ml (Figure 5). Although the proportion of necrotic cells increased proportionally under GFW treatment at 1 mg/ml (Q1), (Figure 5), we observed far fewer necrotic cells than apoptotic cells. Taken together, these data indicate that the major cytotoxic effect of GFW is the induction of apoptosis in bladder cancer cells.

## Discussion

Inadequate outcomes associated with intravesical chemotherapy or Bacillus Calmette-Guerin (BCG) as a supplement to TUR in the treatment of bladder cancer has necessitated the development of alternative approaches to chemo-therapy. Naturally occurring substances, such as those found in traditional Chinese medicine, are promising candidates [19]. Recent studies have established the effectiveness of TCM and naturally occurring substances including Paeonia lactiflora Pall [20], Polyporus polysaccharide [21,22], Lingzhi [23-25] and Cantharidin [26] for chemoprevention as well as the chemotherapy of bladder cancer. However, the applicability of GFW as a chemotherapeutic agent against bladder cancer remains unknown. Previous studies have reported that GFW can improve the condition of stagnated blood, which is related to uterine myomas [11,27], varicocele [28], therosclerosis [29], hypercholesteremia [29] and hot flashes [30,31]. GFW has also demonstrated beneficial effects toward articular inflammation and a protective effect against endothelial dysfunction in patients with rheumatoid arthritis [32]. In terms of its anti-cancer effect, GFW has been shown to inhibit the growth of hepatocellular carcinoma [12,13] and cervical cancer [14]. Moutan Cortex, one of the major components of GFW, has been shown to inhibit proliferation and induce apoptosis of human hepatocellular carcinoma [33]. Our current study demonstrated that GFW can significantly inhibit the proliferation of BFTC 905 cells, obtained from a female grade III stage C bladder carcinoma [34] and TSGH 8301 cells from a male grade II/stage A bladder carcinoma [35]. These results suggest that GFW has similar cytotoxic effects in both of these bladder cancer cell lines obtained from different sexes in different tumor stages. Our results also prove that GFW may be as effective as traditional chemotherapeutic agent, mitomycin C, epirubicin, and cisplatin, in the treatment of bladder cancer. Furthermore, the high selectivity of GFW toward cancer cells greatly reduces the adverse side effects associated with normal bladder urothelium. It is important to note that GFW presents a much higher selectivity index (SI) value than the three mentioned chemotherapeutic agents. Nevertheless, the high value we obtained for GFW (SI >3) [15] warrants further investigation. Intriguingly, according to the definition of SI, the differential SI values of cisplatin to BFTC 905 cells (SI = 0.52) and TSGH 8301 cells (SI = 5.66) suggests that this may involve a different mode of action with regard to these cells [15,36].

Cell cycle analysis results indicate that GFW led to cell cycle arrest in BFTC 905 in the S-phase, while cell cycle arrest in TSGH 8301 occurred in G2/M phase (Figures 4A and 4B). To elucidate the differential effects of GFW on the cell cycle mechanism in these cells, we investigated the expression status of various cell cycle mediators. It was found that GFW treatment resulted in the phosphorylation of CHK2 followed by a substantial expression of P21 (Figure 4C). Through phosphorylation, CHK2 inhibits the function of protein phosphatases CDC25A and CDC25C [37] resulting in the cell cycle arrest at the G1/S and G2/M transitions, respectively [37]. Activated CHK2 can also stabilize P53 [38-41], which subsequently promotes the expression of P21(WAF1) [42]. P21 can thus bind to CDK2-cyclin E complex with a subsequent retardation of kinase activity resulting in the inhibition of cell cycle progression in the S phase [43]. Moreover, P21 can suppress the kinase activities of CDK2-cyclin A and CDK1-cyclin A complexes, which are involved in the cell cycle progression in S and G2 phases, respectively [44]. On the other hand, P21 also affects CDK1-cyclin B1 complex, which is involved in the G2/M transition [44]. Accordingly, we observed a robust increase in P21 in BFTC 905 cells following GFW treatment for 4–6 h (Figure 4C). Cell cycle arrest in the S phase suggests that the kinase activity of CDK2-cyclin E, CDK2-cyclin A, and CDK1-cyclin A complexes were inhibited (Figure 4A). However, GFW appears to have a more complex effect on G2/M cell cycle regulation in TSGH 8301 cells. We observed a substantial basal level of P21 prior to treatment, such that GFW treatment for 6 h provided only a slight, gradual increase in P21 (Figure 4C). These results suggest that a mild increase in P21 is ineffective in inhibiting the kinase activity of CDK2-cyclin E, CDK2-cyclin A, or CDK1-cyclin A complexes for S phase arrest. However, the combined repression of CDC25A and CDC25C by phospho-CHK2 and the partial inhibition of the kinase activity of CDK2-cyclin E, CDK2-cyclin A, CDK1-cyclin A, and CDK1-cyclin B1 could eventually lead to the accumulation of cells in G2/M phase. Thus far, we have only demonstrated the induction of CHK2 phosphorylation, which promotes the expression of P21 in bladder cancer cells treated with GFW. This suggests that cell cycle regulation following GFW treatment might be caused by the profound expression of P21. We suspect that GFW may cause very early genotoxic stress responses in highly replicating cancer cells. These responses may require ATM or ATM-related protein kinase activity for the activation of cell cycle checkpoint factors; however, further investigation is required.

Several studies have shown that cell cycle arrest may lead to the induction of apoptosis [45,46]. Therefore, we sought to determine whether GFW could induce apoptosis in bladder cancer cells. Although we observed that BFTC 905 and TSGH 8301 cells underwent GFW-induced apoptosis in both the early and late stages, most of the apoptotic cells were in the late stage (Figure 5). Treatment with 1 mg/ml of GFW resulted in significantly higher levels of apoptosis than what was observed in the untreated control group, indicating that GFW is a potent inducer of apoptosis in both BFTC 905 and TSGH 8301 cells. However, flow cytometry analysis indicated only a small proportion of necrotic cells in the samples without GFW treatment. The proportion of necrotic cells also underwent a slight increase following an increase in the concentration of GFW. This may be due to apoptotic pathway deficiencies [47] in these cancer cells, in which the DNA damage response pathways may be activated by transiently activating PARP1 to induce necrotic cell death [48].

## Conclusions

Our results indicate that GFW is a potent inhibitor of proliferation in BFTC 905 and TSGH 8301 bladder cancer cell lines. High selectivity to cancer cells and minimal toxicity to normal urothelial cells makes it a good candidate for intravesicle chemotherapy in bladder cancer; however, an in vivo animal study will be required to further validate these results. We have also demonstrated that GFW interferes with cell cycle progression via the activation of CHK2/P21 pathway and induces apoptosis in these bladder cancer cells. GFW may act through specific signaling to bypass defective apoptotic pathways, thereby inducing selective necrotic cell death in these bladder cancer cells. These results warrant further experiments in vitro and in vivo to further elucidate the anti-tumor effects of GFW in bladder cancer.

## Competing interests

The authors declare that they have no competing interests.

## Authors’ contributions

CCL, SYC, and HYH performed experiments. CDH and MWYC performed statistical analysis. CCL, MYL, and CHS participated in the design of the study. MYL and CHS provided all agents used in this study. LGC performed HPLC to determine the components of GFW. CDH and MWYC formulated and directed the study design. All authors have read and approved the final manuscript.

## Pre-publication history

The pre-publication history for this paper can be accessed here:

http://www.biomedcentral.com/1472-6882/13/44/prepub

## Supplementary Material

Additional file 1 Supplemental Information. Click here for file
