# Supplementary material for: The investigation of a traditional Chinese medicine, Guizhi Fuling Wan (GFW) as an intravesical therapeutic agent for urothelial carcinoma of the bladder
Source: BMC Complement Altern Med. 2013 Feb 23;13:44. doi: 10.1186/1472-6882-13-44 (PMC3599278; doi:10.1186/1472-6882-13-44)
Supplement: Additional file 1 — Supplemental Information. [file 1472-6882-13-44-S1.pdf]

Supplemental Information

The Investigation of a Traditional Chinese Medicine, Guizhi Fuling Wan（GFW）as an Intravesical Therapeutic Agent for Urothelial Carcinoma of the Bladder

Chi-Chen Lu, Mei-Yi Lin, Syue-Yi Chen, Cheng-Huang Shen, Lih-Geeng Chen, Hsiao-Yen Hsieh, Michael WY Chan, and Cheng-Da Hsu

Table 1S. Calibration equation for the four compounds: amygdalin, paeoniflorin, cinnamaldehyde, and paeonol

Compound	Calibration equation	Correlation coefficient (R^2)	Concentration range (μg/ml)
Amygdalin	y = 23128x + 32319	0.9998	22~550
Paeoniflorin	y = 22837x - 34855	0.9999	20~1600
Cinnamaldehyde	y = 97244x – 8030.3	1.0000	3.375~45
Paeonol	y = 42210x – 5832.6	0.9995	8~40

n=3.

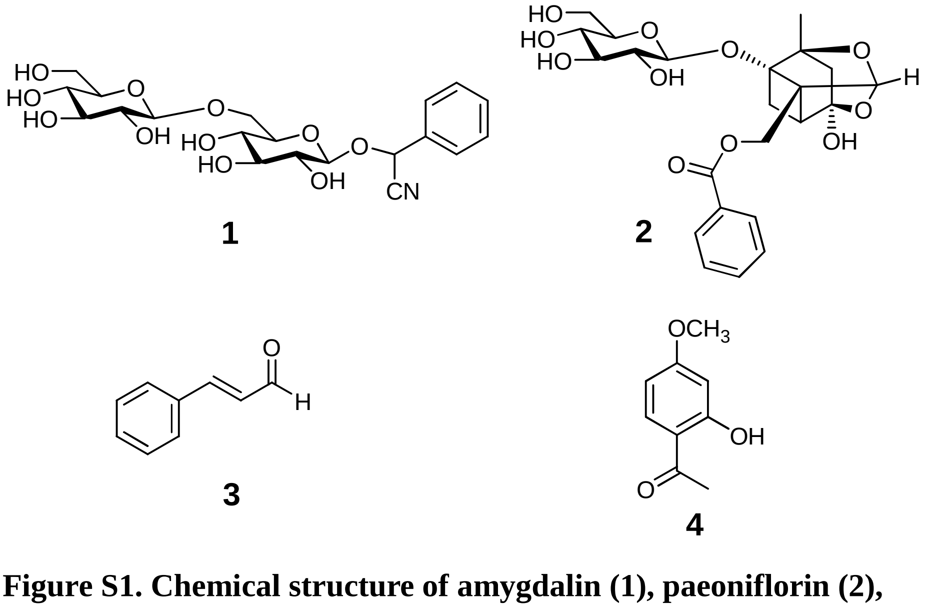

Figure S1. Chemical structure of amygdalin (1), paeoniflorin (2), cinnamaldehyde (3), and paeonol (4) in GFW

Supplemental Experimental Procedures

Sample Preparation

Guizhi Fuling Wan (GFW) herbal extract (batch No. 221141) was purchased from Sun Ten Pharmaceutical Co., Ltd. (Taichung City, Taiwan). Ten grams of GFW was combined in a 50 ml centrifuge tube with 20 ml of double distilled water and shaken at 4 ^{o}C for 48 h. The sample solution was filtered using 0.22 μm PVDF membrane and the filtrate was freeze dried to obtain 1.10 g of powder. The dried powder (46 mg) was combined with 1 ml of 70% methanol and placed in an ultrasonic bath for 30 min. Following centrifugation at 10,000 rpm for 10 min, the supernatant was filtered through a 0.45 μm filter and analyzed by HPLC.

HPLC Analysis of GFW

The HPLC system comprised a LC-10ATvp liquid chromatographic pump, an SIL-10ADvp auto-injector, a CTO-10Avp column oven, a SPD-M10Avp diode array detector, a SCL-10Avp system controller, and Class-VP SP1 software ver. 6.12 (Shimadzu, Tokyo, Japan). HPLC conditions for GFW were as follows: Analysis was performed using a Discovery HS-C18 column (4.6 mm i.d. ×250 mm, 5 μm, Supelco, PA, USA). The mobile phase comprised 0.05% trifluoroacetic acid in water-acetonitrile (0 min, 90:10; 12 min, 90:10; 13 min 88:12; 26 min, 88:12; 27 min,

60:40; 47 min, 60:40; 57 min, 90:10; 58 min, 90:10). The flow rate was 1.0 ml/min and 10 μl portions were injected into the column. The column temperature was set to 40° C. A photodiode array detector detected the compounds in GFW at the following wavelengths: amygdalin (215 nm), paeoniflorin (232 nm), cinnamaldehyde (280 nm), and paeonol (275 nm). The retention times of the compounds were as follows: amygdalin (13.91 min), paeoniflorin (26.31 min), cinnamaldehyde (40.73 min), and paeonol (43.37 min).

Calibration Curve Preparation

Amygdalin (98%), trans-cinnamaldehyde (98%), and paeonol (98%) were purchased from Nacalai Tesque, Inc. (Kyoto, Japan). Paeoniflorin (98%) was purchased from Tauto Biotech (Shanghai, China). The agents were accurately weighed, dissolved, and serially diluted with 70% methanol to give concentrations in the range of 22~550 μg/ml (amygdalin), 20~1600 μg/ml (paeoniflorin), 3.375~45 μg/ml (cinnamaldehyde), and 8~40 μg/ml (paeonol). Calibration curves were plotted following linear regression of the peak areas.

Method Validation

The precision of these methods was evaluated in terms of intra-day and inter-day

assays. The intra-day assay involved triplicate analysis performed within 24 h; inter-day assay involved triplicate analysis performed over 3 days. Every sample was analyzed in triplicate. Average standard deviation (S.D.) and relative standard deviation (RSD) were then calculated.
